# Queries Respecting Vapour Baths

**Published:** 1812-02

**Authors:** 


### *For the Medical and Physical Journal.*

### QUERIES *respecting* VAPOUR BATHS.

1. AS vapour or steam baths have of late been much and deservedly recommended from their powerful and penetrating effects, especially in all *chronic* and obstinate rheumatic affections, it is a much wished-for object to ascertain, whether there is *any* and *what* difference in the vapour of *sea* and that of *fresh* water?

2. Sea-water being rendered fresh or sweet by distillation, does it alter the nature of the steam, so as probably to cause any different effects in its medical application?

3. As two *inflammable gases*, viz. the sulphurated hydrogen, and the ammoniacal gas, are miscible with water, it may be presumed that certain waters and natural springs, impregnated therewith, will have some good effects as medicinal baths; is it not probable that these waters, being reduced into vapour, and applied to medicinal purposes, will act with the same characteristic difference as the simple hot waters and their aeriform substance?

4. In like manner, as the following six uninflammable gases, also miscible with water, viz.

Carbonic acid gas—Muriatic acid gas—
Sulphurous acid gas—Fluoric acid gas—
Phosphoric acid gas, and Nitrous acid gas, exist more or less in natural springs, and, as these waters are frequently applied both as warm-water baths and hot-vapour baths, is it not presumable that these gases will penetrate *diseased parts*, and act with the same characteristic benefit as the simple vapour baths, by solving, dispersing, and expelling, diseased particles, and healing and invigorating the enfeebled organs?

